# TGF-β activates NLRP3 inflammasome by an autocrine production of TGF-β in LX-2 human hepatic stellate cells

**DOI:** 10.1007/s11010-022-04369-5

**Published:** 2022-02-09

**Authors:** Hwansu Kang, Eunhui Seo, Yoon Sin Oh, Hee-Sook Jun

**Affiliations:** 1 College of Pharmacy and Gachon Institute of Pharmaceutical Science, Gachon University, Incheon, Korea; 2 Lee Gil Ya Cancer and Diabetes Institute, College of Pharmacy and Gachon Institute of Pharmaceutical Science, 155 Gaetbeol-ro, Yeonsu-ku, Incheon, 21999 Korea; 3 Department of Food and Nutrition, Eulji University, 553 Sanseong-daero, Sujeong-gu, Seongnam, 13135 Korea; 4 Gachon Medical Research Institute, Gil Hospital, Incheon, Korea

**Keywords:** TGF-β, NLRP3 inflammasome, Hepatic stellate cells, Liver fibrosis

## Abstract

Inflammation contributes to the pathogenesis of liver disease, and inflammasome activation has been identified as a major contributor to the amplification of liver inflammation. Transforming growth factor-beta (TGF-β) is a key regulator of liver physiology, contributing to all stages of liver disease. We investigated whether TGF-β is involved in inflammasome-mediated fibrosis in hepatic stellate cells. Treatment with TGF-β increased priming of NLRP3 inflammasome signaling by increasing NLRP3 levels and activating TAK1-NF-kB signaling. Moreover, TGF-β increased the expression of p-Smad2/3-NOX4 in LX-2 cells and consequently increased ROS content, which is a trigger for NLRP3 inflammasome activation. Elevated expression of NEK7 and active caspase-1 was also shown in TGF-β-induced LX-2 cells, and this level was reduced by (5Z)-oxozeaenol, a TAK inhibitor. Finally, TGF-β-treated cells significantly increased TGF-β secretion levels, and their production was inhibited by IL-1β receptor antagonist treatment. In conclusion, TGF-β may represent an endogenous danger signal to the active NLRP3 inflammasome, by which IL-1β mediates TGF-β expression in an autocrine manner. Therefore, targeting the NLRP3 inflammasome may be a promising approach for the development of therapies for TGF-β-induced liver fibrosis.

## Introduction

The liver plays a role in metabolism and detoxification and is where an active immune response occurs. Liver sinusoids, which are connected directly to the portal circulation, are potent filtering systems against various toxic substances. They contain several cell types, including sinusoidal endothelial cells, Kupffer cells (liver macrophages), and hepatic stellate cells (HSCs). HSCs represent 5%–8% of all liver cells and 13% of sinusoidal cells [1]. HSCs are normally physiologically quiescent, but are rapidly activated during liver injury [2]. Activated HSCs produce and deposit type I and III collagen and fibronectin, and release TGFβ1, a potent fibrotic cytokine, leading to hepatic fibrosis [3]. Therefore, activated HSCs are correlated with fibrosis in various types of acute and chronic liver inflammatory diseases.

The nucleotide-binding oligomerization domain (Nod)**-**like receptor protein 3 (NLRP3) inflammasome is a cytoplasmic multiprotein complex that initiates cell death and triggers the release of inflammatory cytokines IL-1β and IL-18 to induce an inflammatory response [4]. In contrast to other inflammatory responses, inflammasome activation requires two signals: priming (signal 1) and activation (signal 2) [5]. The NLRP3 inflammasome is primed by toll-like receptor (TLR)-4, interleukin-1 receptor (IL-1R), and tumor necrosis factor receptor (TNFR) signaling [6–8]. Priming signaling proceeds through activation of NF-κB following activation of TGF-β-activated kinase 1 (TAK1) [9, 10]. When NF-κB is activated, the expression of NLRP3 and pro-IL-1β increases [11]. The secondary NLRP3 activation step (signal 2) is required at the same time as, or after, the priming step. The activation step is triggered by ATP, pore-forming toxins, viral RNA, or particulate matter. It can proceed through potassium efflux [12, 13], calcium influx [14], intracellular ROS [15, 16] and lysosome rupture [17], thereby promoting NLRP3 inflammasome assembly and caspase-1-mediated IL-1β and IL-18 secretion [8, 18]. Consequently, the IL-1β signaling pathway results in an endogenous increase of TGF-β [19, 20].

The NLRP3 inflammasome is closely related to HSCs activation and is a key mechanism in the regulation of hepatic fibrosis progression [21–23]. In particular, defects in inflammasome-sensing and adaptor molecules have been shown to reduce liver fibrosis by carbon tetrachloride (CCl_4_) and thioacetamide (TAA) [4]. Therefore, understanding the steps involved in NLRP3 inflammasome activation is crucial for the treatment of fibrosis-related liver diseases [16].

The TGF-β signaling pathway activates TAK1-NF-κB signals, which initiate signal 1 for the NLRP3 inflammasome priming step [24–27]. Moreover, TGF-β increases the content of intracellular ROS through the Smad-NOX axis [28–30], which is involved in signal 2 for NLRP3 inflammasome activation. These results suggest that TGF-β may participate in the priming pathway and secondary activation of the NLRP3 inflammasome, but the mechanism of this TGF-β autocrine loop has not been elucidated.

In this study, we investigated whether the NLRP3 inflammasome was activated by TGF-β in HSCs and elucidated the process of TGF-β autocrine loop formation by NLRP3 inflammasome activation.

## Materials and methods

### Cell culture

LX-2 cells (SCC065, Millipore, MA, USA), a human HSC line, were cultured in high-glucose Dulbecco's modified Eagle's medium (DMEM; LM001-70, WelGene, Daegu, Korea) supplemented with 10% fetal bovine serum (FBS; PK004-07, WelGene) and 1% penicillin–streptomycin (P/S; LS202-02, WelGene). Cultured LX-2 cells were starved in 1% FBS-DMEM for 24 h and activated by treatment with 10 ng/mL TGF-β1 (100–21, PEPROTECH, NJ, USA) for 24 h. For the NLRP3 inflammasome activation study, cultured LX-2 cells were starved with serum-free DMEM for 24 h and treated with 10 ng/mL TGF-β1 for 10, 30, and 60 min. Non-treated cells were used as the control. After each incubation time, LX-2 cells were harvested, cultured media were used for ELISA analysis, and the cells were used for protein expression and mRNA expression analysis. The experiment using the TAK1 inhibitor and IL-1R antagonist proceeded as follows. LX-2 cells were untreated (-) or pre-treated with 50 nM of (5Z)-7-oxozeanol or 1 μg/ml of IL-1R antagonist (SRP3327, Sigma) for 1 h and then treated with 10 ng/ml of TGF-β1 for 10 min in the presence of an inhibitor or antagonist. Cell lysates and culture media were prepared for western blotting and ELISA.

### Intracellular ROS contents

The formation of ROS was analyzed using the fluorescent dye 5-(and-6)-chloromethyl-2′,7′-dichlorodihydrofluorescein diacetate, acetyl ester (CM-H_2_DCFDA, Invitrogen, Carlsbad, CA, USA), which is oxidized by intracellular ROS. LX-2 cells (5 × 10^3^ cells/well) were seeded in 96-well black plates (3904, Corning, NY, USA). After 24 h, LX-2 cells were starved with serum-free DMEM for 24 h and treated with 10 ng/mL TGF-β1 for 10, 30, and 60 min. After treatment with TGF-β1, 10 µM H_2_-DCFDA in serum-free DMEM was added to LX-2 cells for 20 min. Treated cells were washed with phosphate buffered saline, and the resulting compound (2,7-dichlorofluorescein, DCF) was measured using a fluorescence microplate reader with an excitation wavelength of 485 nm and an emission wavelength of 535 nm. Intracellular ROS content by TGF-β was expressed as a percentage compared to that in the control group.

### Quantitative reverse transcription PCR

Total RNA in LX-2 cells was isolated for RNA quantification using RNAiso plus reagent (9109, Takara, Otsu, Japan) according to the manufacturer’s protocol. RNA quantification was performed using a spectrophotometer (NANODROP 2000c, Thermo Scientific). Complementary DNA was synthesized from 2 μg RNA using the PrimeScript 1st strand cDNA synthesis kit (6110A, Takara). Quantitative real-time PCR (RT-qPCR) was performed using a reaction mixture of SYBR Premix Ex Taq II (RR82LR, Takara). Specific amplification by PCR was calculated and displayed as a Ct value using a real-time PCR sequence detection system (StepOne Plus, Applied Biosystems, CA, USA). PCR amplification was performed using a two-step method in which the first denaturation was performed at 95 °C for 10 min, followed by 40 cycles at 95 °C for 15 s and 60 °C for 1 min. Relative gene expression was normalized to the housekeeping gene based on the Ct value. The sequences of the primer pairs are shown in Table 1.

**Table 1:** Primer list and sequences for qRT-PCR

Gene	Primer	Oligonucleotide sequences (5'-3')
NLRP3	Forward	GCG TCT GCT GAG GCT CAA GTT A
	Reverse	TTG CTG AGG TAT CGC CAG GAA T
IL-1β	Forward	GAT CGG TGG CTC CAT CCT
	Reverse	CGG CTT CAT CGT ATT CCT GTT
Cyclophilin b	Forward	TGC CAT CGC CAA GGA GTA G
	Reverse	TGC ACA GAC GGT CAC TCA AA

### Western blot analysis

Total proteins were extracted with mammalian extraction buffer (28–9412-79, GE Healthcare, IL, USA) containing a protease inhibitor cocktail (P8340, Sigma), phosphatase inhibitor cocktail 2 (P5726, Sigma), and phosphatase inhibitor cocktail 3 (P0044, Sigma). The extracted proteins (30 μg) were separated by 8%–12% SDS-PAGE and transferred onto nitrocellulose membranes. The membranes were blocked with 5% bovine serum albumin (BSA) or 5% non-fat dry milk, incubated with specific antibodies, and visualized by blotting with horseradish peroxidase-conjugated secondary antibodies. Development was performed using Immobilon Western Chemiluminescent HRP Substrate (WBKLS0500, Millipore, MO, USA). The signals were quantified using the ImageJ software (National Institutes of Health). Antibodies against p-NF-κB (3033), NF-κB (8242), p-TAK1 (4536), Smad2/3 (5678), p-Smad2/3 (8828), β-actin (8457), and TGF-β1 (3711) were used in the experiment and were obtained from Cell Signaling Technology (MA, USA). Antibodies against NLRP3 (NBP2-12,446) were obtained from Novus Biologicals (CO, USA), and those against NADPH oxidase 4 (NOX4, ab195524) were obtained from Abcam. Antibodies against Caspase-1 (ALX-210–804) were obtained from Enzo Life Science (NY, USA) and those against NEK7 (sc-398439), NF-κB (sc-372), TAK1 (sc-7967), IL-1β (sc-52012), TRAF-6 (sc-8409), GAPDH (sc-32233), and horseradish peroxidase-conjugated secondary antibodies were obtained from Santa Cruz Biotechnology Inc.

### Statistical analysis

All data are expressed as the mean ± standard error of at least three independent experiments. Statistical comparisons between two groups were performed using an unpaired two-tailed Student's t-test in Microsoft Excel. To determine the significance among more than two groups, one-way ANOVA was used, followed by GraphPad Prism 7. Statistical significance was set at *p* < 0.05.

## Results

### Treatment of TGF-β activates the NLRP3 inflammasome priming signals in LX-2 cells

**Fig. 1:**
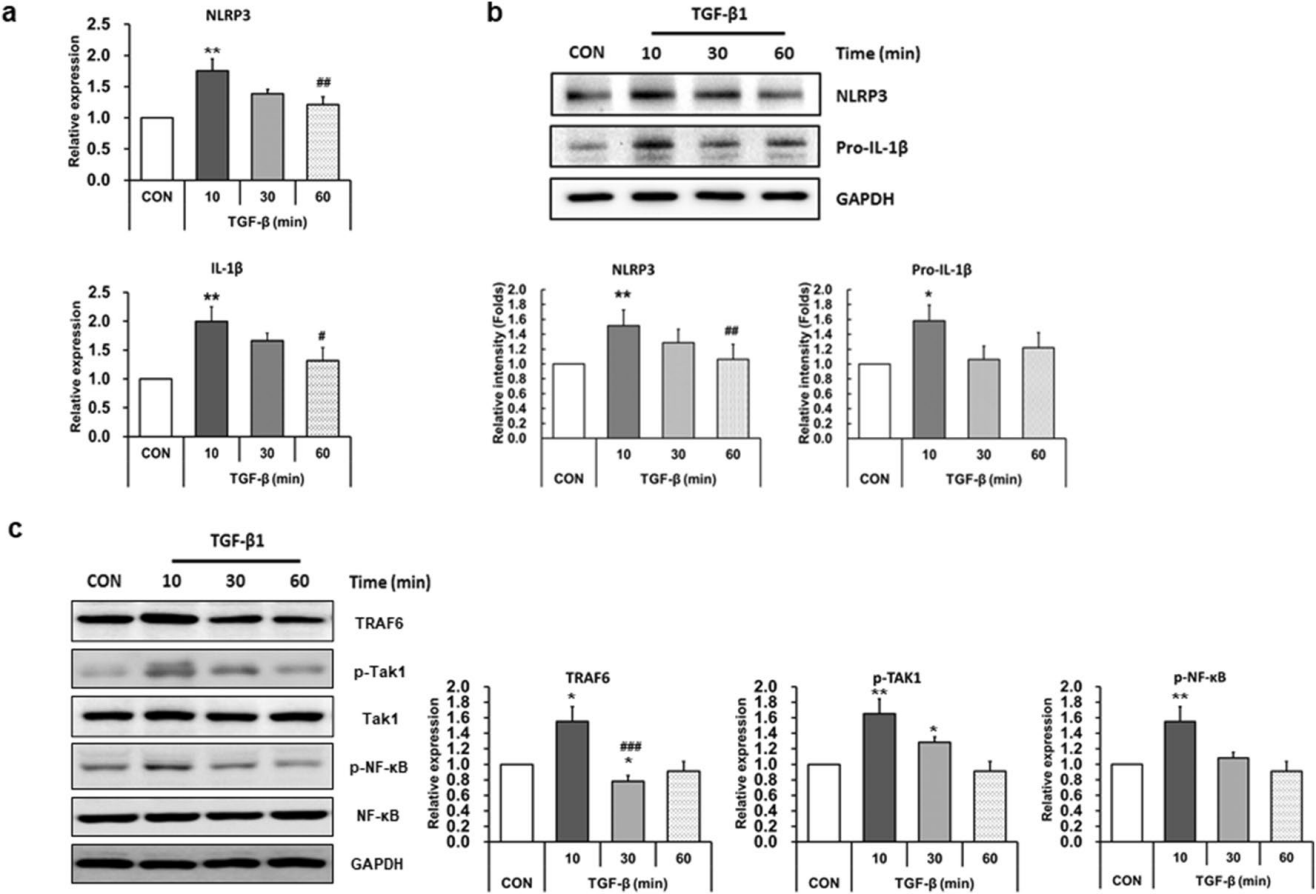
Treatment of TGF-β activates the NLRP3 inflammasome priming signals in LX-2 cells. LX-2 cells were treated with 10 ng/ml TGF-β for 10, 30, or 60 min. **a** mRNA expression levels of NLRP3 and IL-1β were analyzed by quantitative RT-qPCR. mRNA levels were normalized to those of cyclophilin. **b** Cell lysates were prepared and protein levels of NLRP3 and pro-IL-1β were analyzed by western blotting. GAPDH was used as an internal control. Representative western blot (upper panel) and densitometric quantification (lower panel) are shown. **c** The expression levels of priming-associated molecules such as TRAF6, TAK1, and NF-κB were analyzed by western blotting. GAPDH was used as an internal control. Representative western blots (left panel) and densitometric quantification (right panel) are shown. Data are means ± SEM. **p < 0.01, *p < 0.05 *vs.* CON; ^###^*p* < 0.005, ^##^*p* < 0.01, ^#^*p* < 0.05 *vs.* TGF-β (10 min) (*n* = 3/group)

To investigate whether TGF-β increases the NLRP3 inflammasome priming signals, LX-2 cells were treated with 10 ng/ml of TGF-β for various time periods (10, 30, and 60 min), and the expression level of NLRP3 inflammasome-related proteins was examined. As shown in Fig. 1a, mRNA levels of NLRP3 and IL-1β were significantly increased 10 min after TGF-β treatment and reduced at 60 min (Fig. 1a). When we analyzed the protein expression of NLRP3 inflammasome activation from 10 min to 24 h after TGF-β treatment, the level of NLRP3 inflammation-related protein peaked at 10 min. The levels were not significantly changed after 3 h of treatment compared with that in non-treated cells (data not shown). Therefore, we checked the protein expression levels at 10, 30, and 60 min after TGF-β treatment. Protein levels of NLRP3 and pro-IL-1β were also increased 10 min after TGF-β treatment, and the quantification results showed a significant increase at 10 min (Fig. 1b). To confirm the activation of NLRP3 inflammasome priming signals by TGF-β treatment, the upstream targets of IL-1β were investigated under the same conditions. The expression level of TRAF6, a ubiquitin ligase, which is necessary for the priming of NLRP3 inflammasome [31] was significantly increased at 10 min after TGF-β treatment and the level was reduced after 30 min, similar to the non-treated cells (Fig. 1c). Moreover, following the addition of TGF-β, a striking increase in pTAK-1 and p-NF-κB, which is one of the central mediators of the priming signal of NLRP3 inflammasome [32], was detected within 10 min and decreased at 30 min (Fig. 1c), indicating that the NLRP3 inflammasome priming signals were activated by TGF-β treatment.

### Treatment of TGF-β activates the NLRP3 inflammasome activation signals in LX-2 cells

**Fig. 2:**
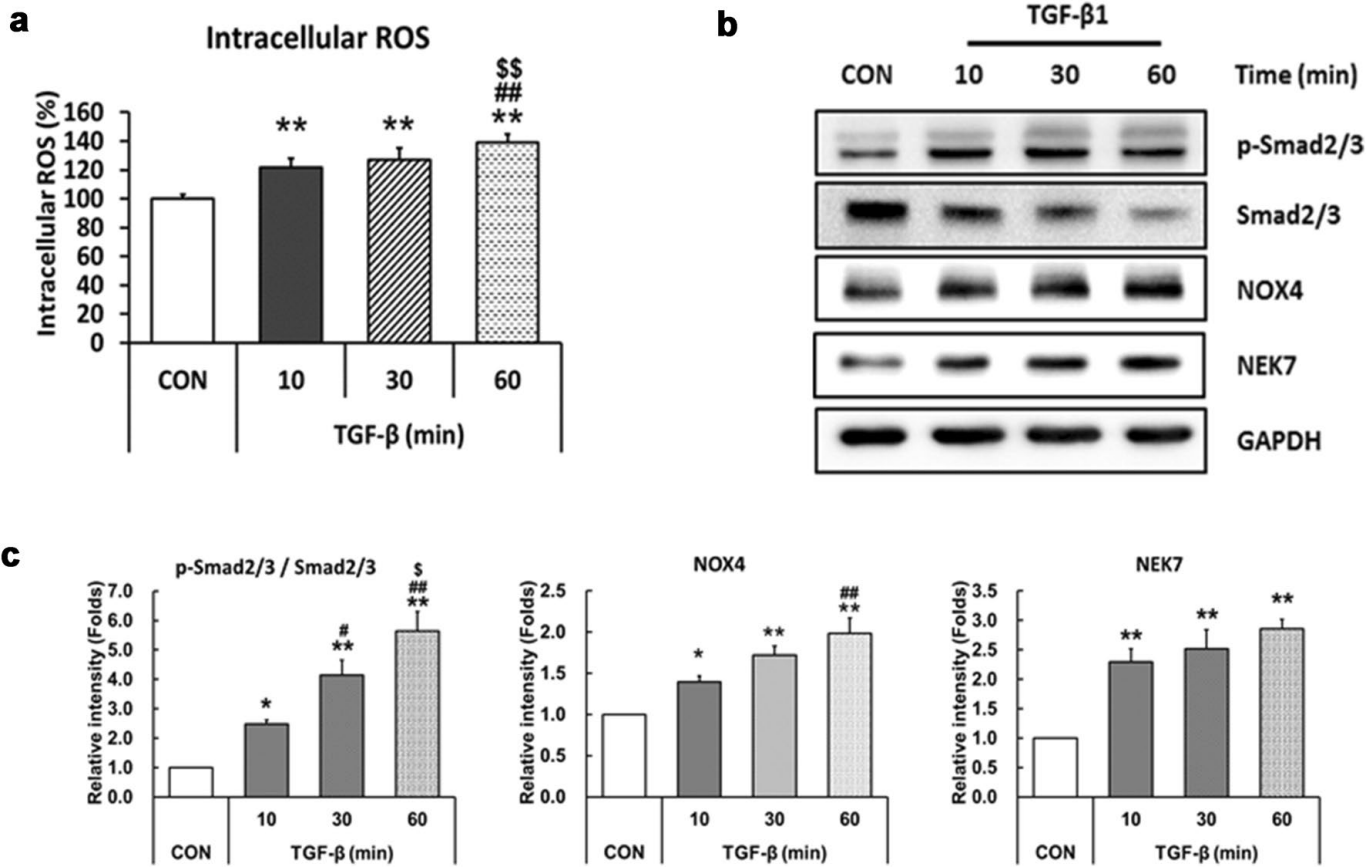
Treatment of TGF-β activates the NLRP3 inflammasome activation signals in LX-2 cells. **a** ROS formation was analyzed by H_2_-DCFDA assay. LX-2 cells were seeded in 96-well black plates and treated with 10 ng/ml TGF-β for 10, 30, or 60 min. After treatment with TGF-β1, the cells were treated with 10 uM H_2_-DCFDA for 20 min. Fluorescence was measured using a microplate reader. **b** LX-2 cells were treated as described in Fig. 2a, and protein levels of p-Smad2/3, smad2/3, NOX4, and NEK7 were analyzed by western blotting. GAPDH was used as an internal control. **c** Densitometric quantification results of western blot images are shown. Data are means ± SEM. **p < 0.01, *p < 0.05 *vs.* CON; ^##^*p* < 0.01, ^#^*p* < 0.05 *vs.* TGF-β (10 min); ^$$^*p* < 0.01, ^$^*p* < 0.05 *vs.* TGF-β (30 min) (n = 3/group)

To determine whether TGF-β activates secondary signals for NLRP3 inflammasome activation, intracellular ROS content was measured using the CM-H_2_DCFDA assay in LX-2 cells. As shown in Fig. 2a, ROS content increased at 10 min after TGF-β treatment and further increased gradually at 60 min (Fig. 2a). As TGF-β increases intracellular ROS content through the Smad-NOX axis [28–30], we measured the expression levels of these signaling molecules. Phosphorylated Smad2/3 was low in cells grown a in serum-free medium and TGF-β-induced Smad2/3 activation within 10 min, reaching peak levels in 60 min. The increase in NOX4 after TGF-β treatment was observed, but the level was slightly delayed compared with p-Smad 2/3. We also found that TGF-β upregulated the level of NEK7, which is a key regulator of NLRP3 inflammasome secondary signaling [33, 34](Fig. 2b, c).

### Treatment of TGF-β activates caspase-1 in LX-2 cells

**Fig. 3:**
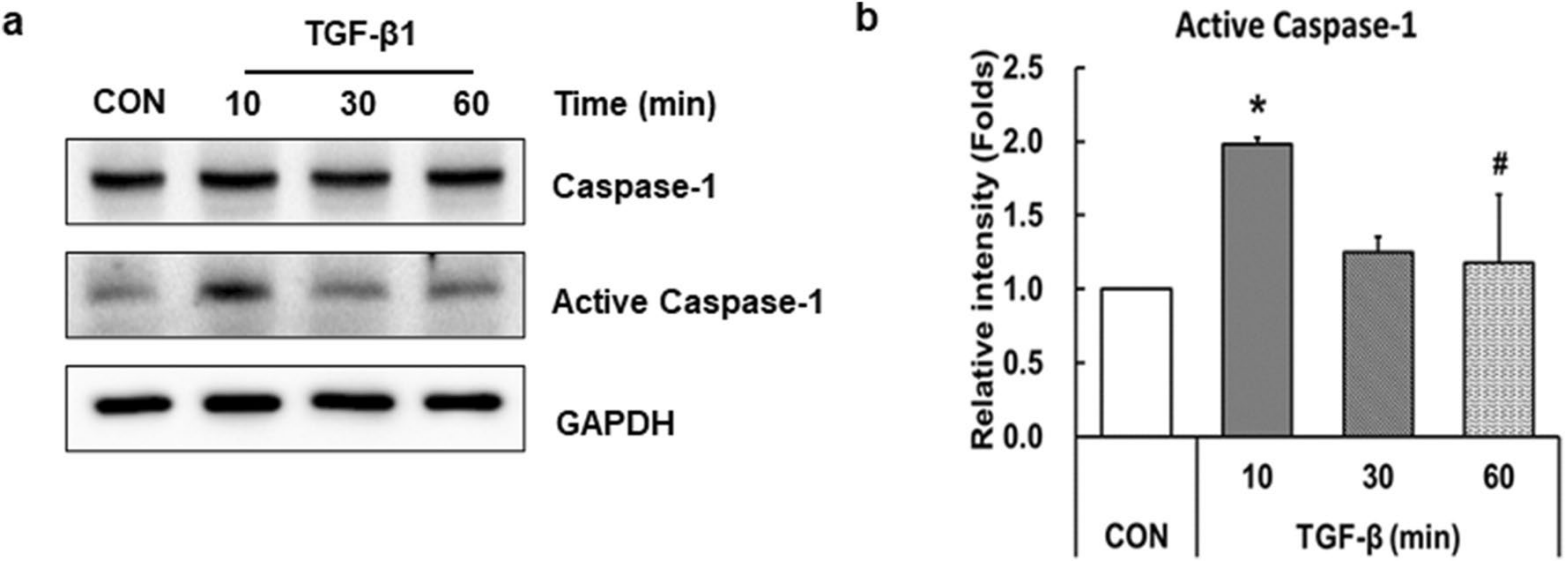
Treatment of TGF-β activates caspase-1 in LX-2 cells. LX-2 cells were treated with 10 ng/ml TGF-β for 10, 30, or 60 min. **a** Cell lysates were prepared, and protein expression was analyzed by western blotting. GAPDH was used as an internal control. **b** Relative levels of active caspase-1 were normalized to GAPDH and quantified using ImageJ software. Data are means ± SEM. *p < 0.05 *vs.* CON; ^#^*p* < 0.05 *vs.* TGF-β (10 min) (*n* = 3/group)

We explored the possibility that TGF-β could activate the NLRP3 inflammasome in LX-2 cells (Figs. 1 and 2). Activation of the NLRP3 inflammasome is known to ultimately increase the active caspase-1 and it cleavages pro-IL-1β to produce the active form of IL-1β [32]. As shown in Fig. 3a, western blot with caspase-1 antibody revealed that LX-2 cells expressed 45 kDa caspase-1 and 20 kDa active caspase-1, and the expression level of active caspase-1 was increased by TGF-β treatment (Fig. 3a). A two-fold increase in protein expression occurred after 10 min of TGF-β treatment, and this level was significantly reduced within 60 min (Fig. 3b). These results indicate that TGF-β activates caspase-1 and induces the secretion of IL-1β in LX-2 cells.

### Treatment of TAK inhibitor ameliorates TGF-β-induced NLRP3 inflammasome priming and activation pathways

**Fig. 4:**
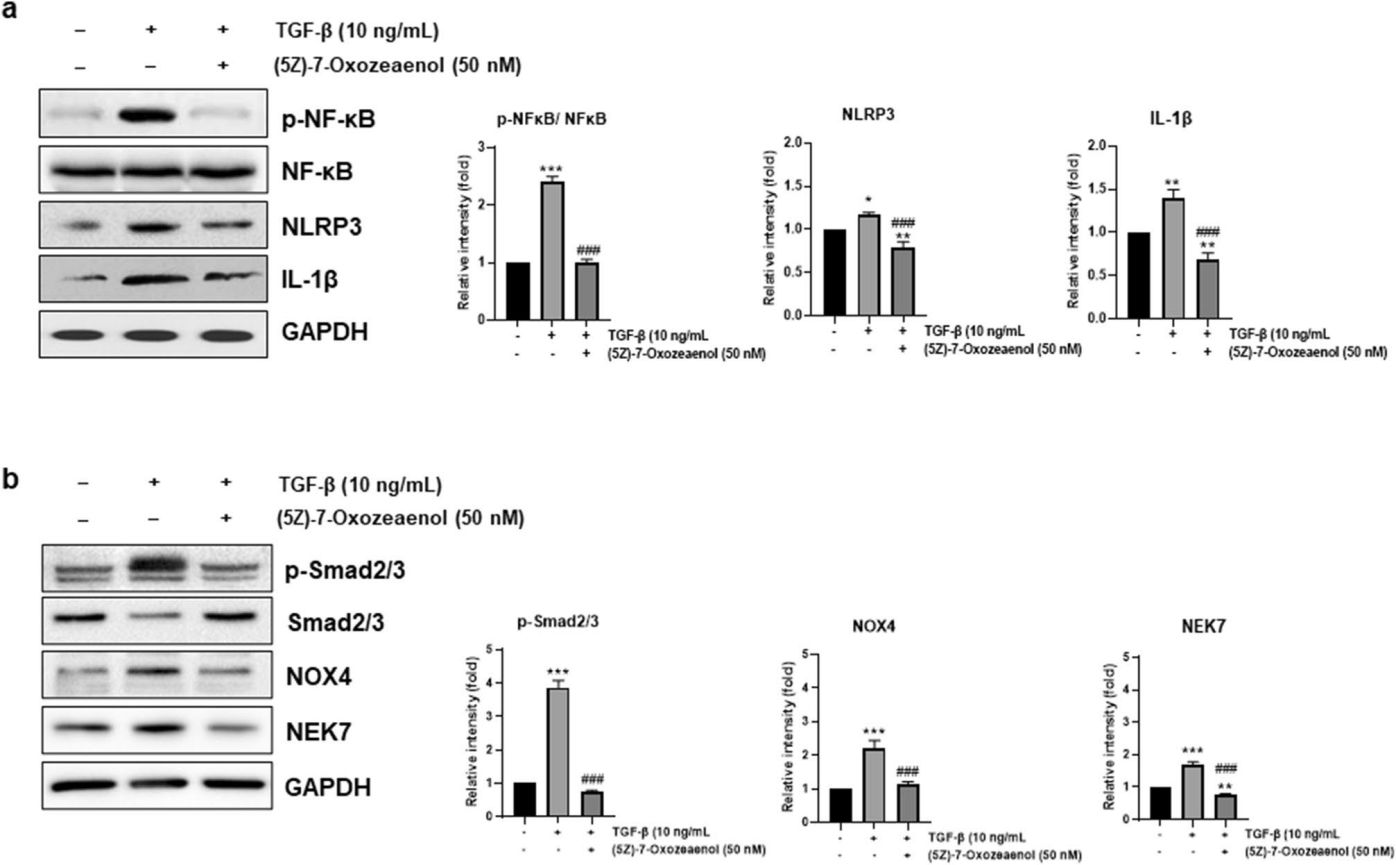
Treatment of TAK inhibitor ameliorates TGF-β-induced NLRP3 inflammasome priming and activation pathways. LX-2 cells were untreated (―) or pre-treated with 50 nM of (5Z)-7-oxozeanol for 1 h and then treated with 10 ng/ml of TGF-β1 for 10 min in the presence of (5Z)-7-oxozeanol. Cell lysates were prepared, and protein expression levels associated with NLRP3 priming and secondary activation were analyzed by western blotting. GAPDH and NF-κB were used as the internal controls. **a** Representative western blot of NLRP3 priming-associated protein (left panel) and densitometric quantification are shown (right panel). **b** Representative western blots of NLRP3 activation-associated protein (left panel) and densitometric quantification are shown (right panel). **p < 0.01, *p < 0.05 *vs.* CON; ^###^*p* < 0.005 *vs.* TGF-β (n = 3/group)

To investigate whether TGF-β-induced NLRP3 inflammasome activation was mediated by TAK1 signaling, (5Z)-7-oxozeaenol, a TAK1 inhibitor, was treated with and without TGF-β, and priming and activation-related protein expression was analyzed by western blot analysis of cell lysates. The TGF-β-induced phosphorylation of NF-κB is shown in Fig. 4a, but treatment with (5Z)-7-oxozeaenol ameliorated the expression of p-NF-kB. Consequently, the expression levels of NLRP3 and active IL-1β, the downstream position of p-NF-kB, were also increased by TGF-β treatment and were significantly reduced by treatment with (5Z)-7-oxozeaenol (Fig. 4a). The protein expression of p-Smad2/3, NOX4, and NEK7, which are related to secondary signals of NLRP3 inflammasome activation, was also increased by TGF-β treatment, but the levels were reduced by TAK inhibitor (Fig. 4b).

### Exogenous TGF-β increases the expression level of TGF-β in LX-2 cells

**Fig. 5:**
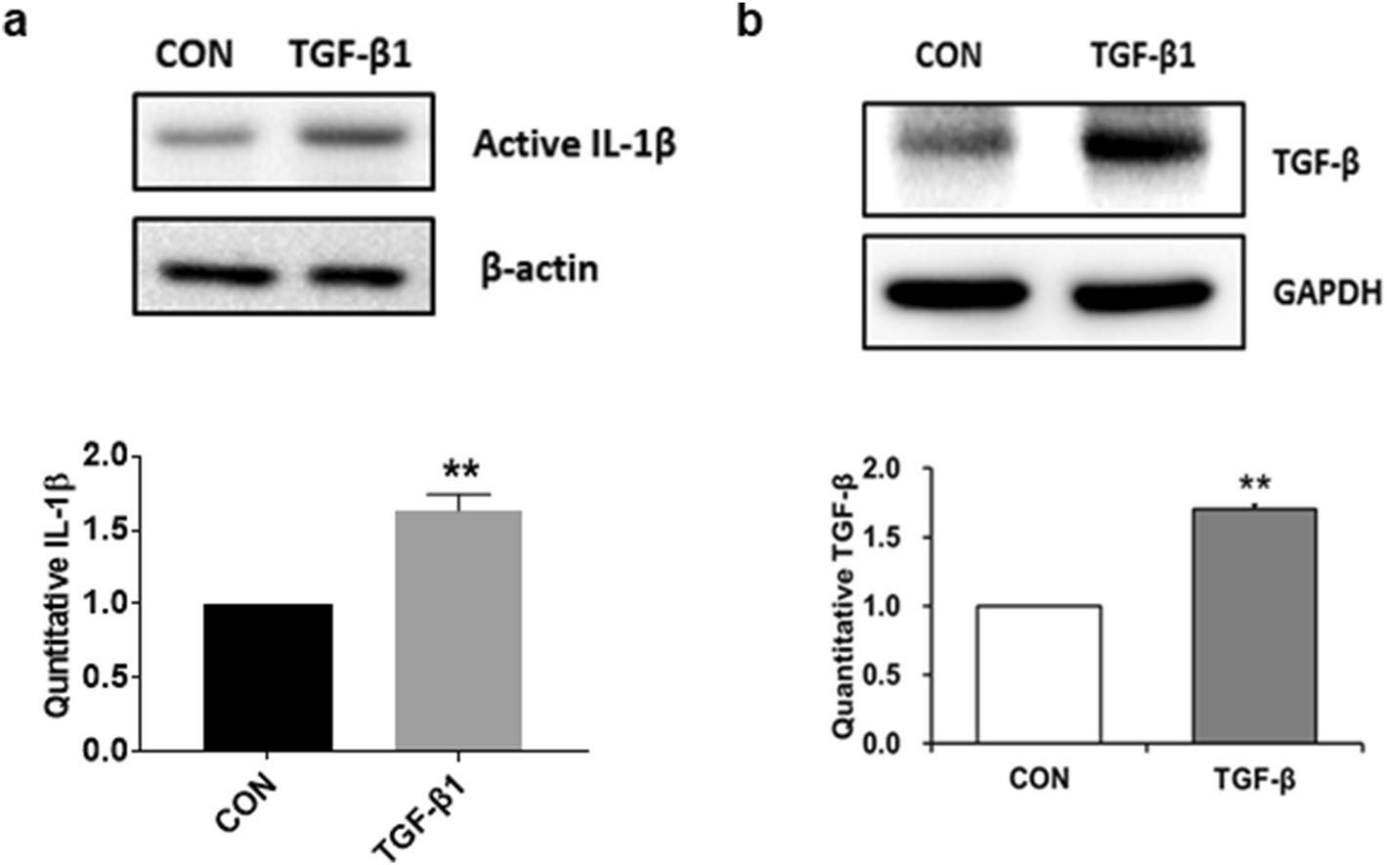
Exogenous TGF-β treatment increases the expression level of TGF-β in LX-2 cells. LX-2 cells were treated with 10 ng/mL TGF-β for 24 h. Cell lysates were prepared and the protein levels of active IL-1β (**a**) and TGF-β (**b**) were analyzed by western blotting. β-actin and GAPDH were used as the internal controls. Representative western blots (upper panel) and densitometric quantification (lower panel) are shown. Data are means ± SEM. **p < 0.01 *vs.* CON. (*n* = 3/group)

NLRP3 inflammasome activation increases endogenous TGF-β expression in HSCs [19, 20]. To investigate whether exogenous TGF-β treatment augmented TGF-β expression via NLRP3 inflammasome pathways, serum-starved cells were incubated in the absence or presence of 10 ng/ml TGF-β for 24 h. Cell lysates treated with TGF-β significantly increased the expression of active IL-1β compared with non-treated cells (Fig. 5a). Moreover, TGF-β expression was increased in response to TGF-β stimulation (Fig. 5b), indicating that TGF-β-induced NLRP3 inflammasome activation acts as an autocrine signaling loop of TGF-β in LX-2 cells.

### Treatment of IL-1R antagonist inhibits TGF-β-mediated TGF-β accumulation in LX-2 cells

**Fig. 6:**
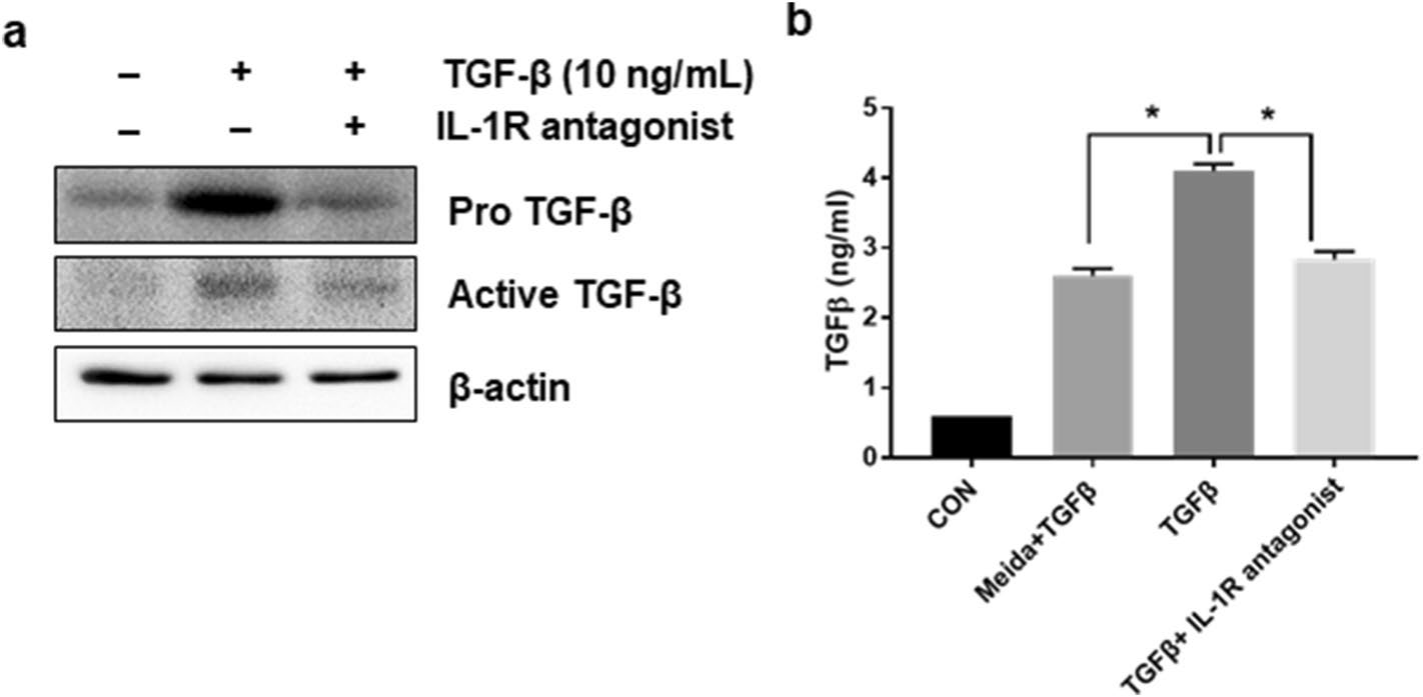
Treatment of IL-1R antagonist inhibits TGF-β-mediated TGF-β accumulation in LX-2 cells. LX-2 cells were untreated (―) or pre-treated with 1 μg/ml of IL-1R antagonist for 1 h and then treated with 10 ng/ml of TGF-β1 for 24 h in the presence of IL-1R antagonist. **a** Cell lysate was prepared, and TGF-β expression was analyzed by western blotting. β-Actin was used as an internal control. **b** The amount of TGF-β released into the supernatant was quantified using an ELISA assay kit and normalized to the amount of total protein. *p < 0.01. (*n* = 3/group)

As we observed that TGF-β increased NLRP3 inflammasome-mediated IL-1β expression (Fig. 1a, b), we next examined whether IL-1β receptor signaling was involved in the process of TGF-β autocrine loop formation by NLRP3 inflammasome activation. Cell lysates and supernatants were prepared, followed by TGF-β for 24 h with and without IL-1R antagonist, and TGF-β levels were examined. Western blotting, as described in Fig. 6a showed that both pro-and active forms of TGF-β were increased by TGF-β treatment, but the levels were dramatically decreased by co-treatment with IL-1R antagonist (Fig. 6a). TGF-β secretion was increased approximately two-fold by TGF-β treatment compared to the TGF-β treatment group without cells, but the levels were significantly decreased by IL-1R antagonist co-treatment (Fig. 6b).

## Discussion

The NLRP3 inflammasome plays an important role in the innate immune response during the development of liver diseases such as non-alcoholic steatohepatitis (NASH), hepatitis, hepatic fibrosis, and liver cancer [35]. It is known that TGF-β is a master profibrogenic cytokine and a promising target to treat fibrosis [36], but a clear mechanism by which TGF-β causes liver fibrosis via the NLRP3 inflammasome is not yet understood. In this study, we found that TGF-β could induce NLRP3 inflammasome activation and subsequent secretion of the potent pro-inflammatory cytokine IL-1β in LX-2 cells. Moreover, IL-1β secreted by TGF-β secretes endogenous TGF-β, which acts in an autocrine manner in HSCs.

The NLRP3 inflammasome priming signal is regulated by receptor-mediated signaling pathways and proceeds through the activation of NF-κB following activation of TAK1 [9, 10]. A number of TGF-β signaling pathways have been identified [37] and the p38 MAPK signaling pathway is involved in the development of fibrosis in animal models of kidney disease [38]. TAK1 is a major upstream signaling molecule in TGF-β receptor-mediated type 1 collagen and fibronectin expression through the activation of MAPK kinase-p38 signaling [37]. In this study, we found that TGF-β increased the protein levels of NLRP3, which was reduced by TAK inhibitor treatment. These results suggest that TGF-β receptor-mediated TAK1-NF-κB signaling is involved in the increased NLRP3 inflammasome priming in LX-2 cells.

NLRP3 inflammasome activation is regulated by ROS production, a process that depends on NOX activation to promote the rapid increase of superoxide-free radicals and H_2_O_2_ in the early recognition of pathogens [39]. Wu et al. reported that treatment of endothelial cells with nicotine-induced cell death and upregulated the expression of NLRP3, caspase-1, and IL-1β; however, it was decreased by pretreatment with the ROS scavenger N-acetyl-cysteine (NAC) [40]. Several studies have demonstrated that TGF-β increases cellular ROS through Smad-NOX4 signaling [28–30] and the TGF-β1/Smad signaling pathway is an important pathogenic mechanism in cardiac, hepatic, pulmonary, and renal fibrosis [41]. It is well-known that TGF-β induces Smad signaling by activating the TGF-β receptor 1 (TβR1). Receptor-regulated Smads are recruited, and subsequent phosphorylation of Smad 2/3 leads them to rapidly interact with Smad4 to transcriptionally activate or repress target genes [41]. In this study, we found that exposure of LX-2 cells to TGF-β results in an increase in phosphorylated Smad 2/3 expression and subsequent increase in NOX-4 and ROS production. These results suggest that ROS production is one of the main pathways for NLRP3 activation by TGF-β treatment in HSCs.

When we checked the involvement of TAK signaling in the TGF-β-mediated inflammasome activation, TAK inhibition ameliorates TGF-β-induced p-Smad2/3, NOX4, and NEK7, suggesting that NLRP3 inflammasome activation, as well as the TGF-β priming step, is a TAK-dependent signaling pathway.

We found that TGF-β–induced inflammasome activation occurred in a very short time (10 min). Consistent with our results, Juliana et al. also reported that incubation with LPS for only 10 min was sufficient to prime the NLRP3 inflammasome in primary macrophages [42]. Although it has been demonstrated that the level of NLRP3 expression is transcriptionally regulated, non-transcriptional regulation (post-translational modification), such as the deubiquitination of NLRP3, also contributes to its activation [43]. Therefore, deubiquitination might be involved in the activation of TGF-β–induced NLRP3 inflammasome activation in LX-2 cells. When we checked the NLRP3 inflammasome activation–related proteins at 24 h after TGF-β treatment, the levels were not significantly changed compared with that in control cells (data not shown). This may be because the increase in signal transduction, which peaked at 10 min, rapidly decreased thereafter. The levels might be repeatedly increased and decreased due to a fluctuation cycle via autocrine secretion of TGF-β. Therefore, an increase in signal transduction was not consistently observed at the time point we checked.

**Fig. 7:**
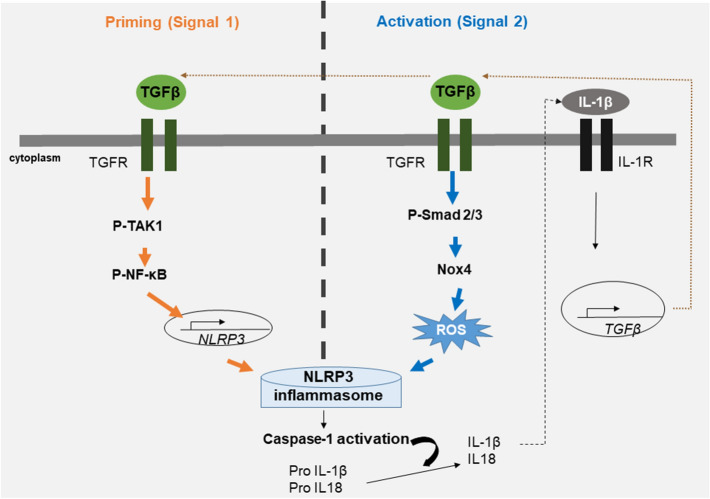
Schematic diagram for the possible mechanism for activation of NLRP3 inflammasome by TGF-β and autocrine loop formation in LX-2 cells. Priming signaling (signal 1) is activated through the TAK1-NF-κB signaling pathway in LX-2 cells by TGF-β. Secondary signaling activation (signal 2) is induced by ROS and is mediated by p-Smad2/3-NOX4 signaling. IL-1β levels are increased by TGF-β-mediated NLRP3 inflammasome activation, and consequently IL-1R signaling pathways involve the accumulation of TGF-β in LX-2 cells. Increased TGF-β acts as an autocrine signaling loop that activates the NLRP3 inflammasome

NLRP3 inflammasome activation increases the secretion of mature IL-1β and leads to increased expression of TGF-β [19, 44]. Increased expression of active IL-1β by TGF-β was confirmed in LX-2 cells, and the cells secrete a high level of TGF-β, which may trigger an autocrine loop. Therefore, a vicious cycle of the TGF-β autocrine loop may promote the progression of liver fibrosis. Moreover, this pathway can be further exacerbated by endogenous danger signals present in the HSC cells, providing TGF-β receptor-mediated priming signals or additional NLRP3 activation signals, such as ROS generation (Fig. 7). A vicious cycle of IL-1β-mediated NLRP3 inflammasome activation has also been observed in atherosclerosis and amyloid β formation in patients with Alzheimer’s disease [45–47]. We found that blockade of IL-1β signaling prohibited TGF-β secretion, demonstrating that IL-1β signaling is necessary for TGF-β secretion by exogenous TGF-β signaling in HSCs.

Taken together, TGF-β may play an important role in hepatic fibrosis progression through the TGF-β autocrine loop by NLRP3 inflammasome activation in LX-2 cells. The autocrine loop as TGF-β-NLRP3 inflammasome-IL-1β-TGF-β could be identified in LX-2 cells, and NLRP3 inflammasome activation can be verified as the key mechanism of the TGF-β autocrine loop in LX-2 cells. Inhibition of TGF-β-related factors or NLRP3 inflammasome may play an important role in breaking the vicious cycle and are recommended as therapeutic targets for the progression and treatment of liver fibrosis.

## Data Availability

The data can be obtained upon request to the corresponding author.
